# Supplementary material for: From sequence to enzyme mechanism using multi-label machine learning
Source: BMC Bioinformatics. 2014 May 19;15:150. doi: 10.1186/1471-2105-15-150 (PMC4229970; doi:10.1186/1471-2105-15-150)
Supplement: Additional file 2 — Java code of ml2db. Additional file ml2db_code.tar.gz contains the Java source code to run the multi-label machine learning experiments and save the results to database. The code’s Javadoc is included. [file 1471-2105-15-150-S2.zip › additional file 2/ml2db/ml2db/doc/main/ml2db/mulan/learn/crosseval/CrossOutputManager.html]

CrossOutputManager


JavaScript is disabled on your browser.


- Overview
- Package
- Class
- Use
- Tree
- Deprecated
- Index
- Help

- Prev Class
- Next Class

- Frames
- No Frames

- All Classes

- Summary:
- Nested |
- Field |
- Constr |
- Method

- Detail:
- Field |
- Constr |
- Method


main.ml2db.mulan.learn.crosseval

## Class CrossOutputManager

- java.lang.Object
- - uk.ac.standrews.utils.main.Initialised
  - - main.ml2db.mulan.learn.crosseval.CrossOutputManager

- All Implemented Interfaces:
  :   uk.ac.standrews.utils.main.Initialisable

  Direct Known Subclasses:
  :   TrainTestOutputManager

  ---

    

  ```
  public class CrossOutputManager
  extends uk.ac.standrews.utils.main.Initialised
  ```

  Abstract class to save the output (evaluation results) of a cross or
  train-test evaluation.

  Version:
  :   14 Jul 2011

  Author:
  :   Luna De Ferrari ldf(at)st-andrews.ac.uk

- - ### Field Summary

    - ### Fields inherited from class uk.ac.standrews.utils.main.Initialised

      `m_initialisationIsCorrect`
  - ### Constructor Summary

    Constructors

    Constructor and Description

    `CrossOutputManager(uk.ac.standrews.utils.main.database.DbManager resultsDbManager,
    java.lang.String resultsFolder,
    uk.ac.standrews.utils.main.database.Table table,
    CrossExperimenter experiment)`
  - ### Method Summary

    Methods

    | Modifier and Type | Method and Description |
    | --- | --- |
    | `java.util.Vector<java.lang.Object[]>` | `getEvaluationMeasures()` |
    | `EvaluationResultsTable` | `getEvaluationResultsTable()` |
    | `uk.ac.standrews.utils.main.database.DbManager` | `getExperimentDbManager()` public MulanCrossEvaluator getEvaluator() { return m\_evaluator; } |
    | `CrossExperimenter` | `getExperimenter()` |
    | `java.lang.String` | `getResultsDirectory()` |
    | `uk.ac.standrews.utils.main.database.Table` | `getTable()` public TableRow getRowValues(Object[] results) { String run = (String) results[0]; int trainArffId = this.getExperimenter().getInputManager() .getTrainArffRecord().getArffId(); // for crossevaluation, same id for training and testing int testArffId = this.getTestId(); String startOfRun = (String) results[1]; String endOfRun = (String) results[2]; MulanLearner learnerName = this.getExperimenter().getLearner(); Evaluation evaluationMetrics = (Evaluation) results[3]; return EvaluationResultsRecord.createEvaluationRecord(run, startOfRun, endOfRun, learnerName, evaluationMetrics, Integer.toString(trainArffId), Integer.toString(testArffId), this.getTable().generateTableManager(this.getExperimentDbManager())); } |
    | `void` | `logEvaluation()` |

    - ### Methods inherited from class uk.ac.standrews.utils.main.Initialised

      `initialisationIsCorrect, setInitialisation, setInitialisationIsCorrect, setInitialisationNotCorrect`
    - ### Methods inherited from class java.lang.Object

      `equals, getClass, hashCode, notify, notifyAll, toString, wait, wait, wait`

- - ### Constructor Detail


    - #### CrossOutputManager

      ```
      public CrossOutputManager(uk.ac.standrews.utils.main.database.DbManager resultsDbManager,
                        java.lang.String resultsFolder,
                        uk.ac.standrews.utils.main.database.Table table,
                        CrossExperimenter experiment)
      ```
  - ### Method Detail


    - #### getEvaluationMeasures

      ```
      public java.util.Vector<java.lang.Object[]> getEvaluationMeasures()
      ```


    - #### getEvaluationResultsTable

      ```
      public EvaluationResultsTable getEvaluationResultsTable()
      ```


    - #### getExperimentDbManager

      ```
      public uk.ac.standrews.utils.main.database.DbManager getExperimentDbManager()
      ```

      public MulanCrossEvaluator getEvaluator() { return m\_evaluator; }


    - #### getExperimenter

      ```
      public CrossExperimenter getExperimenter()
      ```


    - #### getResultsDirectory

      ```
      public java.lang.String getResultsDirectory()
      ```


    - #### getTable

      ```
      public uk.ac.standrews.utils.main.database.Table getTable()
      ```

      public TableRow getRowValues(Object[] results) { String run = (String)
      results[0]; int trainArffId = this.getExperimenter().getInputManager()
      .getTrainArffRecord().getArffId();
      // for crossevaluation, same id for training and testing int testArffId =
      this.getTestId(); String startOfRun = (String) results[1]; String
      endOfRun = (String) results[2]; MulanLearner learnerName =
      this.getExperimenter().getLearner(); Evaluation evaluationMetrics =
      (Evaluation) results[3];
      return EvaluationResultsRecord.createEvaluationRecord(run, startOfRun,
      endOfRun, learnerName, evaluationMetrics, Integer.toString(trainArffId),
      Integer.toString(testArffId),
      this.getTable().generateTableManager(this.getExperimentDbManager())); }


    - #### logEvaluation

      ```
      public void logEvaluation()
      ```


- Overview
- Package
- Class
- Use
- Tree
- Deprecated
- Index
- Help

- Prev Class
- Next Class

- Frames
- No Frames

- All Classes

- Summary:
- Nested |
- Field |
- Constr |
- Method

- Detail:
- Field |
- Constr |
- Method
